# Geriatric Attitude of ChatGPT4.o and Its Evaluation by Social Workers

**DOI:** 10.1093/geroni/igaf122.1181

**Published:** 2025-12-31

**Authors:** Huai Cheng, Jill Vinge

**Affiliations:** Minneapolis VA Hospital, Minneapolis, Minnesota, United States; Minneapolis VA Health System, Minneapolis, Minnesota, United States

## Abstract

Ageism is global and prevalent in healthcare. Previous studies have shown that ChatGPT generated gender and racial biased outputs. This study aimed to explore ChatGPT4.o geriatric attitude using validated UCLA geriatric attitude instrument and to compare it with health trainees and providers and to examine whether hospital social workers agree or disagree with ChatGPT 4.o responses to geriatric attitude statements. Six of 16 statements reflected positive geriatric attitudes, such as “Most old people are pleasant to be with”. Ten statements reflected negative geriatric attitudes, such as “Treatment of chronically ill old patients is hopeless”. ChatGPT4.o responses and social workers’ evaluation of ChatGPT4.o responses were graded on a Likert scale of 1-5 (1=strongly disagree, 5 =strongly agree). Positive and negative geriatric attitudes scores were calculated to compare them to the medical students, ED residents, and neurologists from the previously published studies. The positive geriatric attitude score by ChatGPT4.o was 4.2 and higher than humans (higher scores indicated a more positive geriatric attitude), which was agreed by 40 social workers as 4.1 on a Likert scale of 1-5. The negative geriatric attitude score by ChatGPT4.o was 1.8 and lower than humans (lower scores indicated a less biased aging attitude), which was slightly agreed by 40 social workers as 3.2 on a scale of 1-5. In conclusion, ChatGPT4.o had a better geriatric attitude than humans, which is consistent with our previous findings using ChatGPT3.5. Social workers had modest agreement with ChatGPT4.o geriatric attitude. It suggests ChatGPT4.o is less likely to generate age-biased outputs.

